# Epigenetic Alterations in Density Selected Human Spermatozoa for Assisted Reproduction

**DOI:** 10.1371/journal.pone.0145585

**Published:** 2015-12-28

**Authors:** Bolan Yu, Hua Zhou, Min Liu, Ting Zheng, Lu Jiang, Mei Zhao, Xiaoxie Xu, Zhaofeng Huang

**Affiliations:** 1 Key Laboratory of Reproduction and Genetics of Guangdong Higher Education Institutes, Third Affiliated Hospital of Guangzhou Medical University, Guangzhou, 510150, China; 2 Key Laboratory for Major Obstetric Diseases of Guangdong Province, Third Affiliated Hospital of Guangzhou Medical University, Guangzhou, 510150, China; 3 Institute of Human Virology, Zhongshan School of Medicine, Sun Yat-sen University, Guangzhou 510080, China; 4 Department of Biochemistry, Zhongshan School of Medicine, Sun Yat-sen University, Guangzhou 510080, China; 5 Key Laboratory of Tropical Diseases Control (Sun Yat-sen University), Ministry of Education in China, Guangzhou 510080, China; University of Bonn, Institut of experimental hematology and transfusion medicine, GERMANY

## Abstract

Epidemiological evidence indicates that assisted reproductive technologies (ART) may be associated with several epigenetic diseases such as Beckwith-Wiedemann syndrome (BWS) or Silver-Russell syndrome (SRS). Selection of sperm by density-gradients in ART has improved DNA integrity and sperm quality; however, epigenetic alterations associated with this approach are largely unknown. In the present study, we investigated DNA methylation and histone retention profiles in raw sperm and selected sperm derived from the same individual and separated by using density-gradients. Results from a study group consisting of 93 males demonstrated that both global DNA methylation and histone retention levels decreased in density selected sperm. Compared to unselected raw sperm, histone transition rates decreased by an average of 27.2% in selected sperm, and the global methylation rate was 3.8% in unselected sperm and 3.3% in the selected sperm. DNA methylation and histone retention location profiling analyses suggested that these alterations displayed specific location patterns in the human genome. Changes in the pattern of hypomethylation largely occurred in transcriptional factor gene families such as *HOX*, *FOX*, and *GATA*. Histone retention increased in 67 genes, whereas it was significantly clustered in neural development-related gene families, particularly the olfactory sensor gene family. Although a causative relationship could not be established, the results of the present study suggest the possibility that sperm with good density also possess unique epigenetic profiles, particularly for genes involved in neural and olfactory development. As increasing evidence demonstrates that epigenetics plays a key role in embryonic development and offspring growth characteristics, the specific epigenetic alterations we observed in selected sperm may influence the transcriptional process and neural development in embryos.

## Introduction

Sperm separation is routinely utilized in the clinic for assisted reproductive technologies (ART)such as intrauterine insemination (IUI) and *in-vitro* fertilization (IVF). Using density-gradients, a high percentage of morphologically normal and motile human spermatozoa can be isolated from seminal plasma, and these are also free of debris, dead spermatozoa, and non-germ cells[1]. During this process, the epigenetic profiles including histone retention and DNA methylome could be potentially altered, except for DNA integrity. Several studies have shown that children born by ART have a higher risk for imprinting disorders such as Beckwith-Wiedemann syndrome (BWS) or Silver-Russell syndrome (SRS) [2–4].However, the epigenetic alterations resulting from sperm separation for ARTremains largely unknown.

Histone to protamine transition is considered a key step in the establishment of epigenetic stability in spermatozoa. One rare phenomenon during spermatogenesis is that most histones in sperm are replaced by protamines[5]. Protamines are rich in arginines that have a net positive charge and cysteine residues can form multiple disulfide bonds, making DNA packaging in sperm about 6 times more compact than that observed in somatic cells [6]. In human sperm, the histone to protamine exchange rate has been estimated to be around 85% [7]. Abnormalities in histone transition and protamine expression in humans may be associated with defective spermatogenesis, male infertility, and failed ART[8–12]. For instance, elevated histone to protamine ratios and altered protamine expression are widespread in infertile men[9, 10], and these abnormalities have been linked to a decrease in successful fertilization in ART[11–13].

DNA methylation is another epigenetic process during spermatogenesis that also affects the clinical results of ART and is linked with embryonic development. Previous reports have shown that the pregnancy rate is higher when sperm DNA methylation is above a certain threshold[14, 15]. Infertile men, especial oligozoospermic patients, often undergo aberrant DNA methylation of imprinted loci in sperm,which in turn can be passed on to their offspring[16]. In addition, in normazoospermic men, hypomethylation of the H19 gene has been linked to recurrent abortion[17]. Therefore, the abnormal epigenetic information carried by spermatozoa that have prepared for ARTmight also be transmitted to the next generation.

Recent research investigations demonstrate that environmentally induced parental epigenetic alterations can be passed into the next generations and influence the characteristics of the offspring [18–22]. In addition, CpG-rich sequences in the mouse genome packages genes for embryonic development and serve as the molecular determinants of sperm nucleosome retention[23, 24]. However, information on the epigenetic profile of dense sperm selected for ART is limited. Have histone retention and DNA methylome changed after sperm are selected by density-gradients? Are there any specific or special alterations in selected sperm? Is there any linkage between DNA methylome alterations and histone retaining sites? These questions are not only important for the analysis of epigenetic alterations in ART, but also relevant to fertilization and embryology.

To address these questions, we separated 93semen samples from either normzoospermic men or oligoasthenozoospermic patients using density-gradients. DNA fragmentation, histone to protamine transition, and genome-wide DNA methylation in spermatozoa were analyzed before and after semen separation. Using MeDIP(methylated DNA immunoprecipitation)-chip array and high-throughput sequencing, specific alterations in DNA methylation and histone retention were also analyzed. The findings of the present study have demonstrated that both profiles had changed in density selected sperm that were separated from raw sperm, which in turn serve as novel evidence for specific alterations in the DNA methylome and histone retention in ART.

## Materials and Methods

### Study subjects

The present study was approved by the institutional review board of the Third Affiliated Hospital of Guangzhou Medical University and performed in compliance with the Helsinki Declaration (2008). Study participants were ethnic Han Chinese from Guangzhou City and its surrounding regions in southern China. The participants were scheduled for interviews after providing written informed consent. The interviewers collected data regarding medical history, lifestyle, and smoking status via a structured questionnaire. Men who were unhealthy or whose defective spermatogenesis had a known cause such as varicocele, infection, obstruction of the vas deferens, chromosomal abnormalities, or microdeletions in the azoospermia factor region on the Y chromosome, were excluded. Patients who were diagnosed with severe oligozoospermia, azoospermia, hemospermia, leukospermia, and necrozoospermia were also excluded. Finally, semen samples from 93 individuals were used, of which 39 were normozoospermic males, and 54 were asthenozoospermic or oligoasthenozoospermic patients.

### Sperm collection and determination of clinical parameters

Semen samples from patients were collected in sterile containers by masturbation after 2–7 days of sexual abstinence. Samples were allowed to liquefy for at least 30 min at room temperature. Analyses of semen volume, pH, sperm concentration, vitality, and motility, and computer-assisted semen analysis (CASA) were conducted according to WHO standards [1]. To avoid somatic cell contamination, every specimen of raw sperm were examined under microscopy, and only those samples with sperm cells more than 99.5% were further used in this study.

### Sperm preparation by discontinuous density-gradients

Each sample was isolated and purified using a standard gradient isolation procedure issued by the WHO [1]. Briefly, a density-gradient medium was prepared in a 15-mL test-tube by layering 1 mL of 40% (v/v) density-gradient medium over 1 mL of 80% (v/v) density-gradient medium (In vitro, Denmark). About 1 mL of each well-mixed semen sample was placed on top of the density-gradient media and then centrifuged at 400*g* for 20 min. Most of the supernatant was removed from the sperm pellet, which was then resuspended in 5 mL of supplemented medium by gentle pipetting. The separated sperm pellet was centrifuged at 400*g* for 10 min twice, and the final sperm pellet was resuspended in 0.5 mL of supplemented medium. After the clinic parameters of separated sperm were analyzed, both separated sperm solution and the remaining portion of the raw semen was centrifuged at 1,000*g* for 10 min and stored as a cell pellet at -80°C.

### Aniline blue staining

The ratio of histone to protamine in sperm nuclei was measured using a Nucleoprotein Transition Test Kit (Huakang Company, Shenzhen, China). Briefly, 5 μL of prepared spermatozoa were spread across a glass slide and allowed to dry. The smears were fixed in 3% buffered glutaraldehyde in 0.2 M phosphate buffer (pH 7.2) for 30 min. The slides were then stained with 5% aqueous aniline blue mixed with 4% acetic acid (pH 3.5) for 5 min. Positive staining was indicative of abnormal histone to protamine transition. A total of 400 sperm cells were evaluated under a microscope and the percentage of stained sperm heads was calculated.

### Sperm chromatin dispersion assay

The Sperm chromatin dispersion assay(SCD) assay was performed according to manufacturer’s guidelines(Huakang Company, Shenzhen, China). Briefly, fresh semen samples were diluted in PBS to a concentration of 10 × 10^6^/mL. At 37°C, 30 μL of the diluted sample was added to 30 mL of 1% low melting point agarose and thoroughly mixed. About 20 μL of the semen agarose solution was pipetted onto slides precoated with 0.65% agarose and covered with a 22×22 mm coverslip. Slides were then placed on a cold plate at 4°C for 5 min to allow the samples to gel. Slide covers were then removed, and the slides were immediately immersed horizontally in a denaturation solution for 7 min at room temperature, and then horizontally immersed in a lysis solution for 20 min. Slides were then washed with distilled water for 5 min, followed by sequential dehydration in 70%, 90%, and 100% ethanol for 2 min each. Slides were allowed to air dry, and covered with Rey’s dye for 10 min. Finally, the slides were washed with tap water and air dried for observation. A minimum of 400 sperm per sample were then scored under the 200× objective lens and halos were scored as large, medium-size, small, or absent. Sperm with small or absent halos were considered to have incurred DNA damages, and SCD results were expressed as the percentage of sperm with fragmented DNA.

### Chromatin isolation and sequencing of the histone-associated DNA

Chromatin isolation was performed following the protocol described elsewhere [25]. Briefly, sperm is washed with PBS twice and resuspended in a lysis buffer containing NP-40 and sodium deoxycholate. MNase was added to digest genomic DNA without the protection of histones. The soluble chromatin fraction was isolated after centrifugation. DNA was obtained by phenol/chloroform extraction after treatment with RNaseA and proteinase K. The DNA samples were size-selected by 5% agarose gel electrophoresis to ensure the use of mononucleosomal DNA (~150 bp in length) in the preparation of sequencing libraries. Illumina sequencing platforms Hiseq2500 system with a PE125 pattern was used in high-throughput sequencing (Novogene Co., Beijing, China).

### Analysis of the data generated by high-throughput sequencing

Low-quality reads and adaptor sequences were filtered from the raw data using the manufacturer’s software (Illumina workstudio, San Diego, CA, USA) to generate clean data. All clean data were qualified by FastQC software and aligned to the hg18 genome by using the R package Rsubread[26, 27]. Differential peaks between paired samples before and after separation were detected by MACS2 [28]. Consistent alterations in all samples were identified using the Bedtools software package[29], and peak2gene in Cistrome pipeline platform was used to find neighboring genes of different peaks (http://cistrome.org/ap/root). Reads alignment on the genome were standardized and counted using IGVtools and snapshots of regions of interest were captured using the IGV browser [30, 31].

### Gene ontology and KEGG pathway analysis

Gene Ontology (GO) term and pathway analysis was performed with DAVID bioinformatics website platform using default parameters [32, 33]. Gene classes were identified based on Gene Ontology term categories and the KEGG pathway database.

### Global DNA methylation analysis

Global DNA methylation analysis was conducted using MethylFlash Methylated DNA Quantification kit (Epigentek Group Inc., Farmingdale, NY, USA). Briefly, 100 ng of isolated sperm DNA from selected samples was used.The whole ELISA process was conducted according to the protocol recommended by the manufacture. The global methylation of each samples were conductedin triplicates and the final value is their average. A standard curve was generated using negative and positive internal controls in the same plate, and the amount of methylated DNA were calculated according to the formula provided in the manufacturer’s protocol.

### Methylation profiling by chip array and data analysis

DNA methylation profiling was performed using MeDIP-chip assay with NimbleGen Human DNA Methylation 3x720 K Promoter Plus CpG Island Array chip by KangChen Co.(Shanghai, China) following manufacture guideline (NimbleGene, Madison, WI, USA). Raw data was extracted as pair files by NimbleScan software. Median centering, quartile normalization, and linear smoothing were conducted using the Bioconductor packages Ringo, limma, and MEDME, respectively. Peak-finding algorithm is provided by NimbleScan v2.5 (Roche-NimbleGen). A one-sided Kolmogorov-Smirnov (KS) test is applied for peak finding following manufacture’s guide. Peaks within 500bp of each other are merged. All peak intensity log2-ration means were transformed to z-scores with a mean = 0 and SD = 1. Cluster analysis was performed with the parameters of Euclidean distance and average linkage clustering. The DEPs called by the NimbleScan algorithm. Identified enriched peaks were mapped to the hg18 genome. Genes that displayed significantly different methylation patterns before and after sperm separation in all three paired samples were selected for the generation of a heatmap, which was drawn using an R package pheatmap.2.

### Statistical analysis

The unpaired t test was used to analyze the normally distributed, numerical data of two groups. The paired *t* test was used for statistical analysis between methylation rates before and after density-gradients of each subject. KS normality test and *F* test was used to test if the data were normally distributed and had equal variance. All analyses were performed using GraphPad Prism 4.0 for Windows (GraphPad Software, CA, USA) and a two-sided P value of 0.05 was considered statistically significant.

## Results

### 1. Demographic and clinical data of study subjects

Age and basic semen parameters of the subjects are shown in Table 1. In a total of 93 males, 52 patients presented with defective spermatogenesis and 41 controls generated normazoospermic semen. There were no significant differences in age, semen volume, and sperm vitality between the cases and the controls (Table 1). However, the patients had significantly lower sperm concentrations, sperm counts, and sperm motility than that in the controls (*P*<0.01, Table 1).

**Table 1:** Demographics and semen parameters of study subjects.

Parameters	Subjects	Controls	Patients
Number	93	41	52
Age(yrs)	32.9±5.1 ^a^	33.6 ± 4.8	32.4 ± 5.2
Sperm concentration (10^6^ cells/mL)	42.8 ± 34.1	60.0 ± 42.2	29.3 ± 16.1*
Sperm volume (mL)	3.7 ± 1.3	3.6 ± 1.2	3.8 ± 1.4
Sperm count (10^6^ cells per ejaculate)	146 ± 106	194 ± 106	108 ± 89*
Sperm progressive motility (%)	40 ± 15	52 ± 11	30 ± 11*
Sperm vitality (%)	82 ± 8	83 ± 8	81 ± 8

^a^: Data are presented as mean ± SD.

*: *P*<0.01 compared to the controls.

### 2. Sperm parameters, histone transition, and DNA fragmentation in raw sperm and density selected sperm

Sperm motility, sperm DNA fragmentation, and sperm nuclear histone to protamine transition were assayed inspermatozoa in raw sperm and selected sperm from the same individual. The results showed that after sperm density-gradient centrifugation, several sperm parameters had improved in the 3 groups, including all subjects, controls, and cases (Table 2). In all subjects, sperm DNA fragmentation decreased from 21.0% to 15.0%, abnormalities in histone transition decreased from 17.3% to 12.6%, and sperm motility increased from 40% to 56% (Table 2). In normazoospermic semen (controls), sperm DNA fragmentation decreased from 16.0% to 10.6%, the abnormality in histone transition decreased from 16.3% to 10.6%, and sperm motility increased from 52% to 66% (Table 2). In abnormal semen (patients), sperm DNA fragmentation decreased from 24.9% to 18.5%, abnormalities in histone transition decreased from 18.0% to 14.1%, and sperm motility increased from 31% to 48% (Table 2). All parameters of selected sperm and raw sperm in 3 groups were significantly different with *P*<0.05 or *P*<0.01. The correlation coefficients of sperm motility and histone transition or DNA fragmentation were calculated before and after sperm separation. The results showed that both sperm DNA fragmentation and histone to protamine transition were strongly associated with sperm motility; before sperm separation, the *P* value was 0.001 and 0.039, respectively, whereas after separation, the *P* value was 0.015 and 0.000, respectively.

**Table 2:** DNA fragmentation and histone transition in raw sperm and selected sperm.

	All subjects n = 93		Controls n = 41		Patients n = 52	
	Raw	Selected	Raw	Selected	Raw	Selected
Sperm motility	40±15	56±16*	52 ±11	66±10*	31 ±11	48±16*
DNA fragmentation	21.0 ± 14.1	15.0 ± 12.8*	16.0±12.7	10.6 ± 7.1*	24.9 ± 13.9	18.5 ±15.1*
Histone transition abnormality	17.3 ± 12.5	12.6 ± 12.1*	16.3 ±13.9	10.6 ± 9.8^#^	18.0 ± 11.1	14.1 ± 13.4*

#: *P*<0.05

*: *P*<0.01 compared to the data of raw sperm.

### 3. Alterations in global methylation in selected spermatozoa

**Fig 1:**
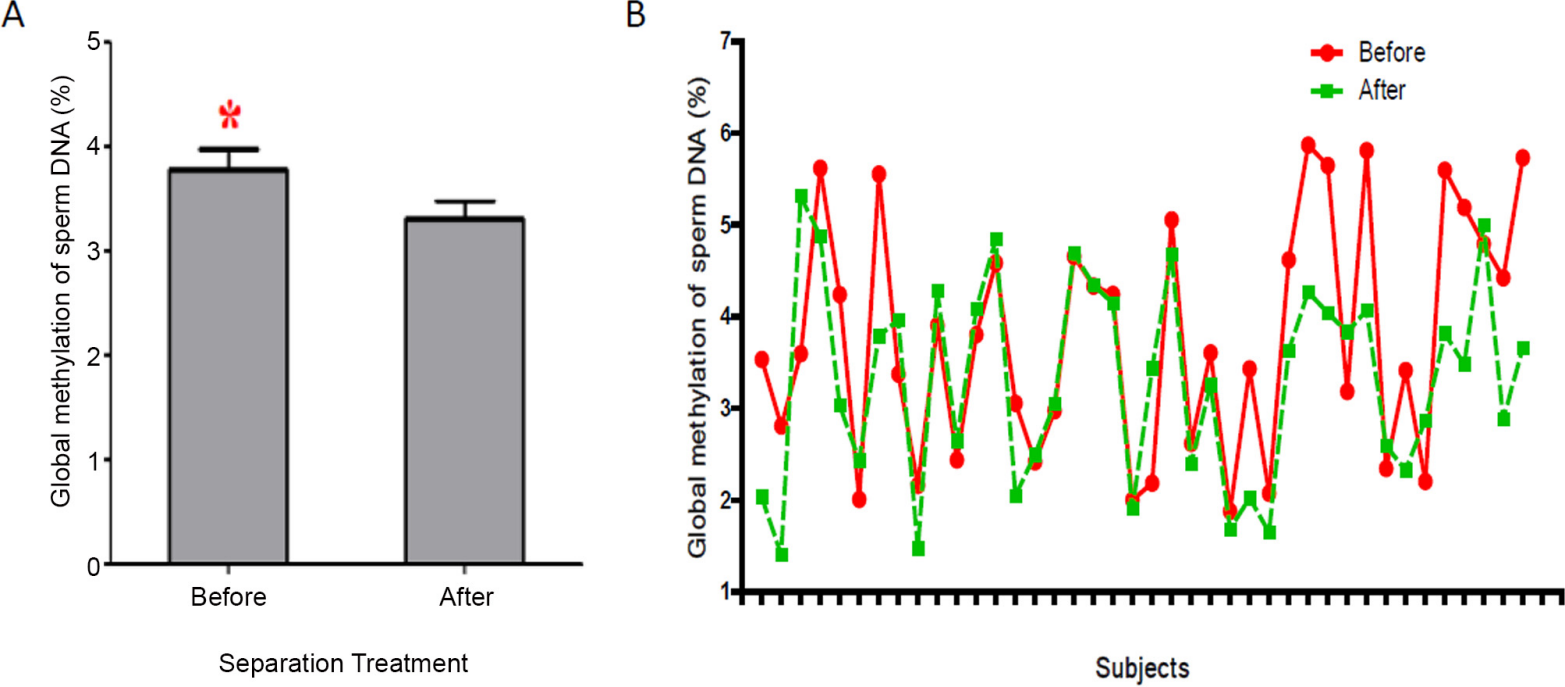
Global methylation alterations in raw sperm and selected sperm. (A)Boxes show alterations in global methylation levels. (B) Individual alterationsing lobal methylation levels.

Using ELISA, the total methylation level in raw sperm and selected sperm from the same individual was measured. Due to the low DNA concentration of some samples, only 45 subjects were analyzed. Intra-individual comparisons indicated significant alterations in global methylation in samples after density-gradients (Fig 1). The relative global methylation rate of raw sperm before separation was 3.8%, whereas that of selected sperm after separation was 3.3%, with a significant decrease of 15% on average (*P*<0.05, Fig 1A). In addition, most subjects showed a decrease in global methylation rate after density-gradients (Fig 1B).

### 4. Alterations in methylation profiles in spermatozoa after separation

**Fig 2:**
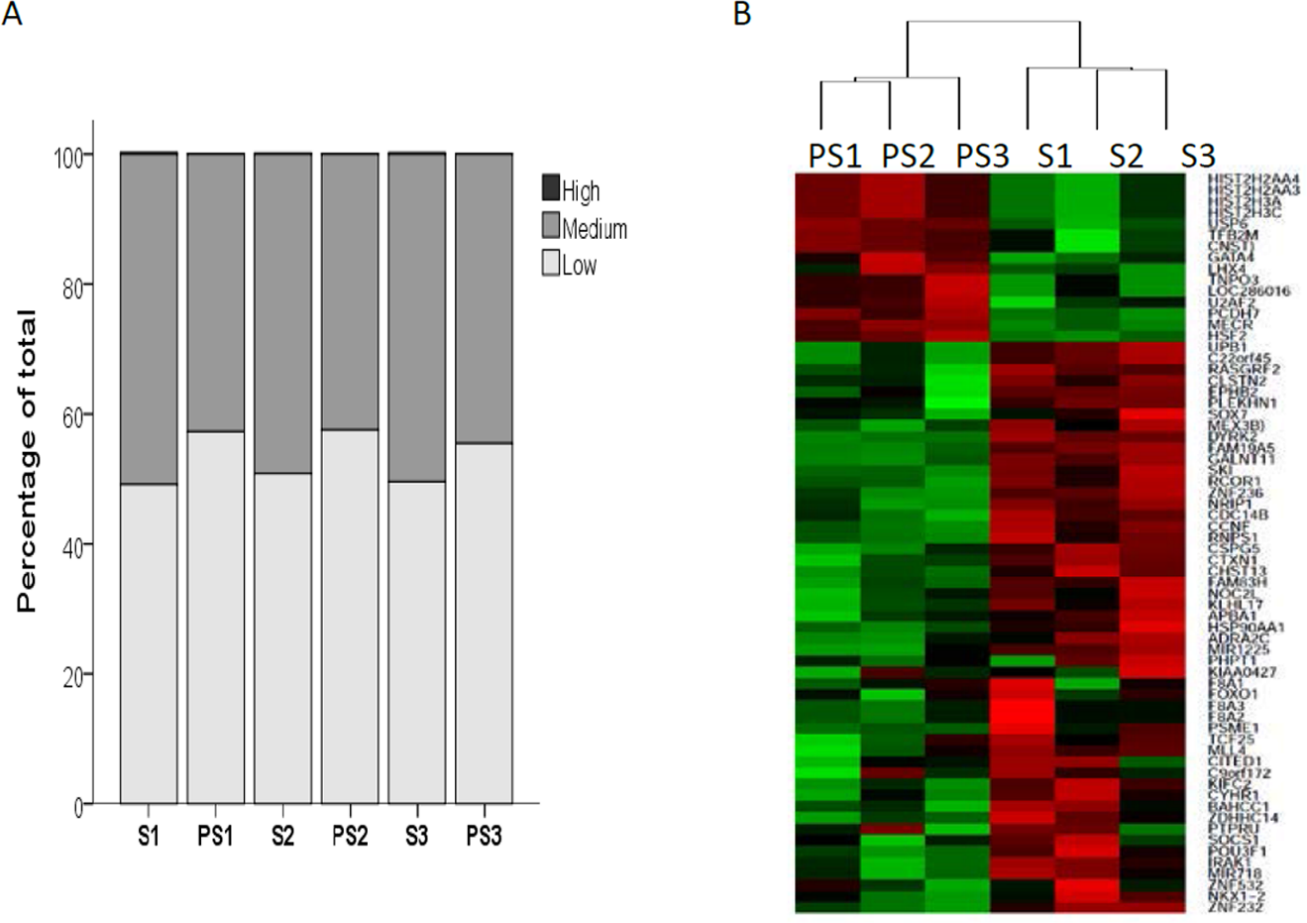
Methylation alterations in paired sperm samples. (A) Fraction of methylation values in raw and selected spermatozoa: S1–S3: Spermatozoa in raw semen from 3 patients. PS1–PS3: selected spermatozoa after density-gradient separation in 3 patients. High: methylation in CpG island sites with M values>2; Medium: methylation in CpG island sites with M values>1; Low: methylation in CpG island sites with M values<1. (B)Hierarchical clustering and heatmap of methylation patterns in spermatozoa: Samples were clustered according to the methylation peak data. Columns represent samples, and rows indicateenriched peak correlated genes. A heat map showing relative methylation differences among all samples. Red indicates high degree of methylation, where as green represents low degree of methylation.

Three pairs of representative samples(raw sperm and density selected sperm, respectively) were further analyzed to determine their DNA methylation profiles using a MeDIP-chip array. Assay results identified 16,760, 14,353, and 14,124 enriched methylation peaks in the sperm genome of raw sperm and 14,206, 14,651, and 14,776 peaks in selected sperm. The global profiles of CpG methylation showed that all 3 patients incurred similar methylation alterations after semen processing (Fig 2A). The number of high-methylation sites was very low (<0.5%) in all 3 samples. The medium methylation sites were 50.85%, 49.17%, and 50.44% in 3 fresh samples, but were 42.70%, 42.45%, and 44.54% in 3 processed counterparts. The low methylation sites were 49.15%, 50.83%, and 49.56% in 3 fresh samples, but were 57.29%, 57.55%, and 55.46% in their processed counterparts. Analysis of promoter subcategories, promoters with high, intermediate, and low CG islands, as well as intergenic and intragenic CG regions demonstrated similar results, which indicated low methylation sites status in spermatozoa after separation (Fig 2A). Clustering analysis was performed with all identified enriched peaks, which identified two branches that correlated well with the semen separation process (Fig 2B).

### 5. Specific gene methylation alterations in selected spermatozoa after separation

Normalized log2-ratio data of each sample were further analyzed based on enrichment peaks. Differentially enriched peaks were associated with 79 genes in all three paired samples with similar patterns of alterations. 68 genes IDs could be identified in DAVID Bioinformatics database and gene ontology(GO) analysis of these genes indicated that they were strongly enriched(p value < 0.05)in several representative GO term which related with gene expression regulation, apoptosis, cell communication, signals and receptors, (Table 3).

**Table 3:** Gene ontology analysis shows gene cluster in selected sperm using density-gradients.

GO Term	Hypomethylated	Hypermethylated	Enrichment fold	EASE Score
GO:0010468~regulation of gene expression	RCOR1, ZNF232, ZNF532, ZNF236, POU3F1, TCF25, NKX1-2, FOXO1, CITED1, NRIP1, SOX7, IRAK1,APBA1, KIAA0427, SKI, SQSTM1, PTPRU	GATA4, LHX4, TFB2M,HSF2	2.118247	5.72E-04
GO:0006916~anti-apoptosis	IRAK1, SQSTM1, LHX4, FOXO1		5.593818	0.032768
GO:0010646~regulation of cell communication	IRAK1, SQSTM1, CITED1, SKI, USP6,SOCS1,EPHB2, RASGRF2,ADRA2C,CSPG5		2.775353	0.007513
GO:0060395~SMAD protein signal transduction	SKI, CITED1		96.02721	0.020233
GO:0048513~organ development	SOCS1, CCNF, FOXO1, POU3F1, PTPRU, FAM83H, TCF25, CITED1, EPHB2, NRIP1	GATA4, LHX4	1.989056	0.028841
GO:0048731~system development	CCNF, SOCS1, FOXO1, CSPG5, PTPRU, CITED1, NRIP1, EPHB2, POU3F1, FAM83H, TCF25, APBA1	GATA4, LHX4	1.730963	0.0435
GO:0007275~multicellular organismal development	SOCS1, CCNF, FOXO1, SKI, CSPG5, PTPRU, CITED1, NRIP1, EPHB2, NKX1-2, POU3F1, FAM83H, TCF25, APBA1	GATA4, LHX4	1.608833	0.0487
microRNA	MIR718, MIR940, MIR1225			
other	ZDHHC14, DYRK2, HSP90AA1, PSME1, RNPS1, BAHCC1, KLHL17, C22orf45, FAM19A5, UROC1, CLSTN2, SQSTM1, GALNT11, CYHR1, PHPT1, CDC14B, F8A1	MEX3B, GP1BB, U2AF2, CNST,TNPO3, PCDH7, HIST2H2AA3		

### 6. Alteration of histone retention loci after separation

A total of 49.2 and 58.0 million reads were respectively obtained from paired samples before and after density-gradient separation of subject 1(S1 and PS1), and56.0 and 51.2 million reads from subject 2(S2 and PS2), 41.7 and 43.6 million reads from subject 3(S3 and PS3). After mapped to genome hg18, different peaks after density-gradient selection were called out with MACS2. Among these different peaks, 2,300 positive peaks and 5,537 negative peaks were displayed with similar pattern in all three paired samples. 67 genes were identified as nearby positive peaks and 1,362 genes as nearby negative peaks in 5kb region window.

**Fig 3:**
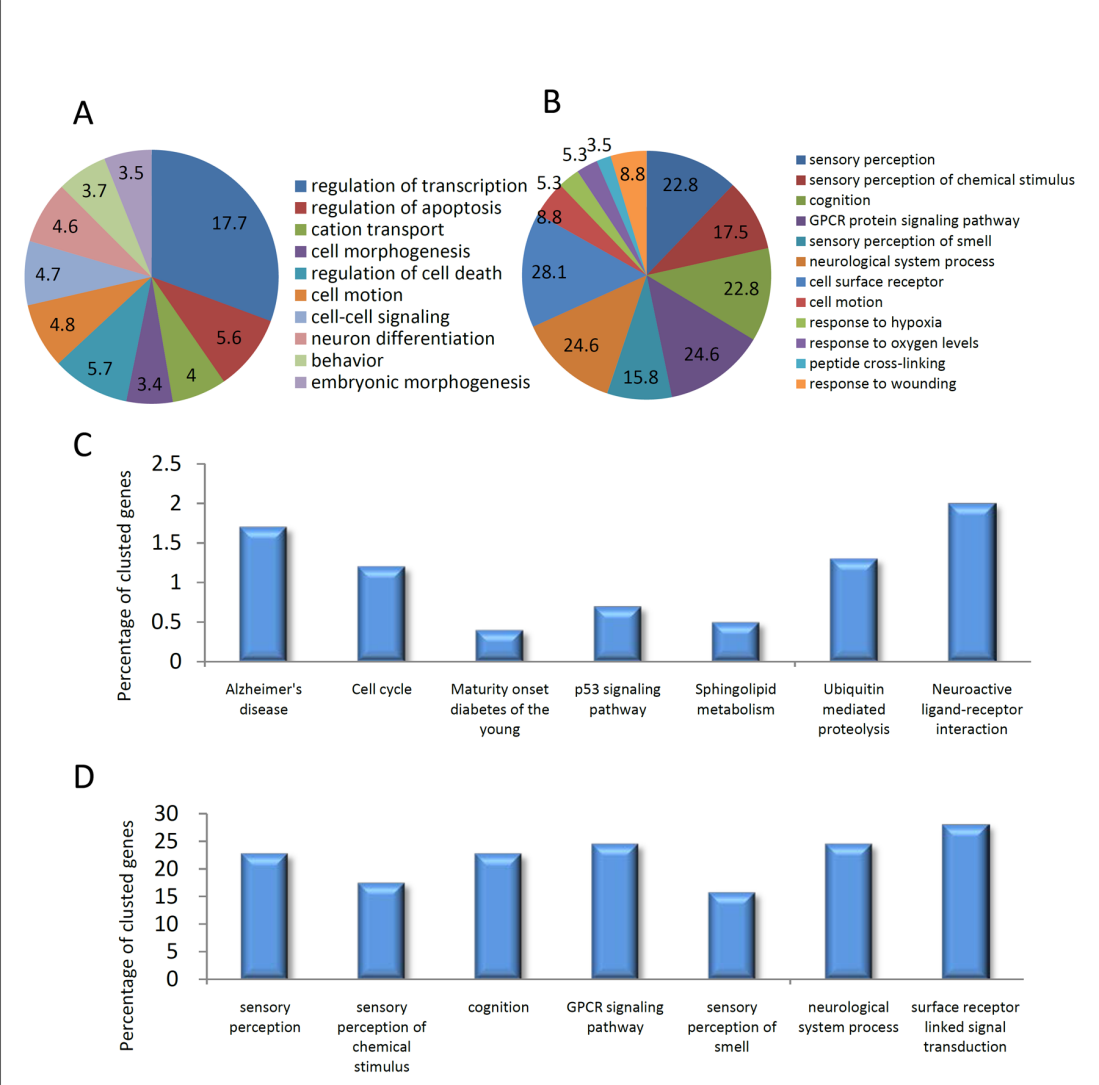
Specific genes with altered histone retention in dense sperm. **(**A) Ten representative enriched GO terms with highest counts in negative peak-related genes in dense sperm. (B)Enriched GO terms in positive peak-related genes in dense sperm.(C) Enriched KEGG pathways in negative peak-related genes in dense sperm. (D) Enriched GO terms in positive peak-related genes in dense sperm with a *P* value < 0.05.

**Fig 4:**
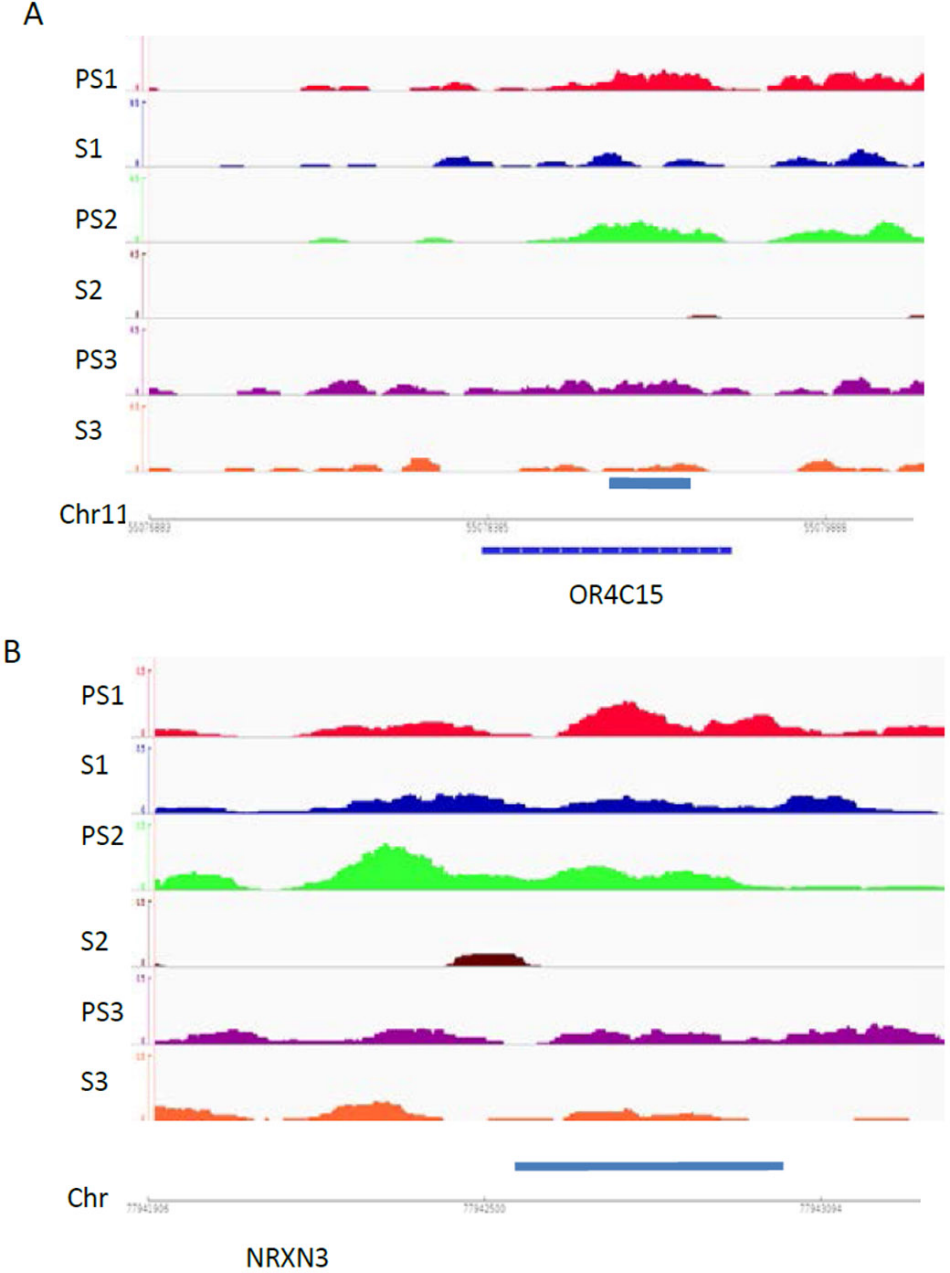
Histone retention alterations in selected genes. Snapshot showing nucleosome profiles of 3 paired samples, denoted as PS1 and S1, PS2 and S2, and PS3 and S3 at OR4C15 (A)and NRXN3(B).Images represent read counts per base. Read counts were standardized for total counts/1 million reads in each sample.

These genes were further analyzed by GO and pathway cluster analyses.67 positive peak-related genes could be enriched to 12 GO terms and 8 GO term clusters(Tables A and B in S1 File).The enriched 12 GO terms are shown in Fig 3B, with count percentage in all GO genes, and 7 of these with *P* values< 0.05 are shown in Fig 3D.A total of 1,362 negative peak-related genes could also be enriched to 259 GO terms and 117 GO term clusters (Tables C and D in S1 File), and 10 representative enriched GO terms are shown in Fig 3A.Using the KEGG pathway database, 9 positive peak-related genes could be enriched to the olfactory transduction pathway(Table E in S1 File), which were also shown in GO and GO cluster-enriched terms (Fig 3B and Table A in S1 File). Negative peak-related genes could be enriched in nine KEGG pathways (Table F in S1 File and Fig 3C) and four KEGG pathway clusters (Table G in S1 File). In the positive peak-related genes, neural development related genes such as cell surface receptor, G-protein coupled receptor, neurological system process, sensory perception, cognition, and sensory perception of chemical stimulus were specially enrichment(Table A in S1 File). Among these, NRXN3 and OR4C15 are representative genes that showed a decrease in histone retention in selected dense sperm (Fig 4).

### 7. Correlation between DNA methylation and histone retention alteration in spermatozoa after separation

**Fig 5:**
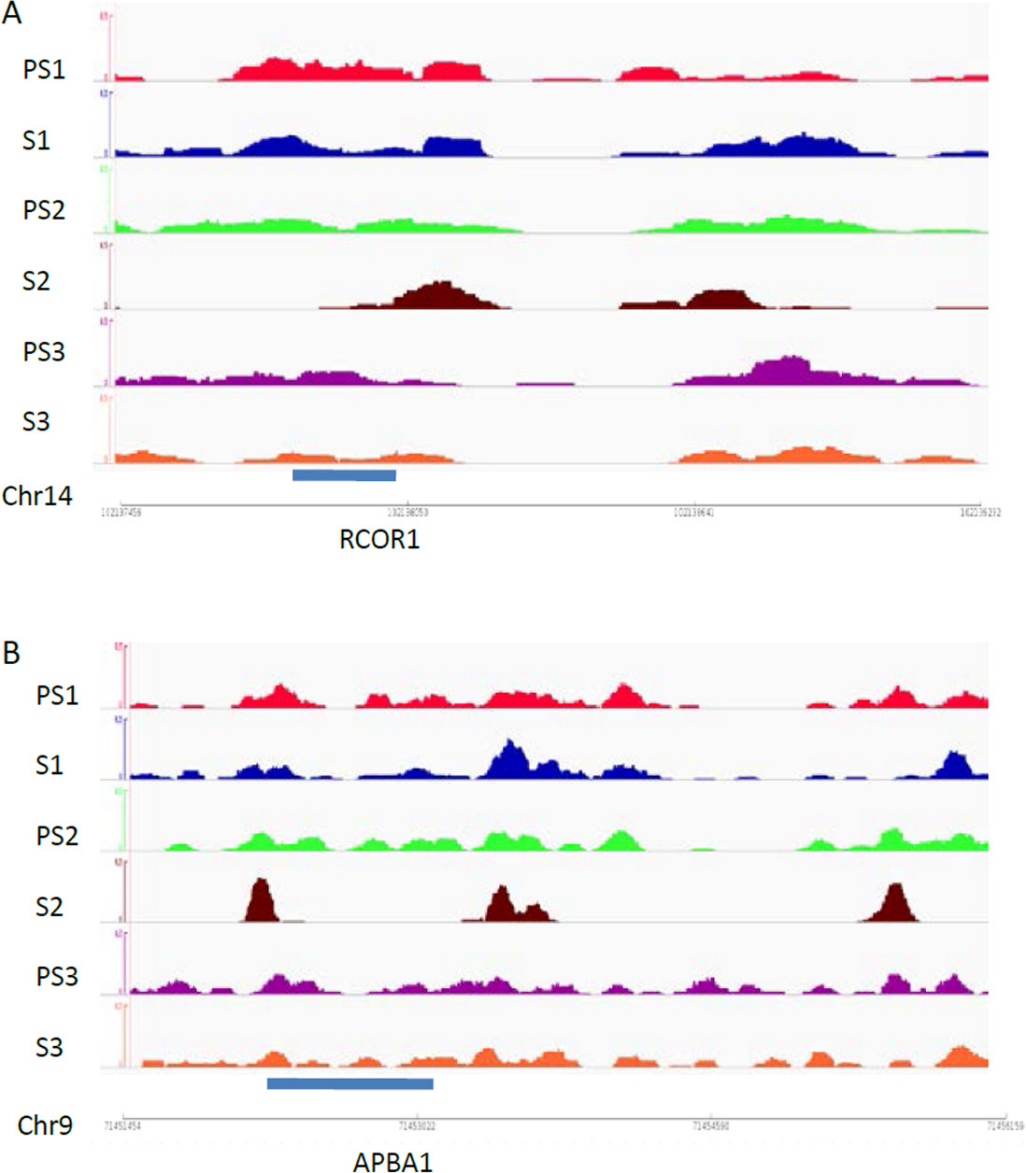
Interaction between DNA methylation and histone retention of genes. Snapshot showing nucleosome profiles of 3 paired samples, denoted as PS1 and S1, PS2 and S2,and PS3 and S3 at RCOR1 and APBA1. Images represent read counts per base. Read counts were standardized for total counts/1 million reads in each sample.

Analysis of DNA methylation profiles and histone retention alterations using the Cistrome pipeline platform identified two sites that were located in the same genes, namely RCOR1 and APBA1,respectively (Fig 5). Both of these alteration sites showed increased histone retention and decreased methylation in selected spermatozoa for ART.

## Discussion

The results of the present study demonstrated that sperm separation using density-gradients significantly increased sperm quality, including sperm motility, sperm morphology, and DNA fragmentation (Table 2). These results are consisted with previous report that sperm DNA fragmentation decreases after sperm processing with swim-up, density-gradients, and combination methods [34], and DNA integrity also improves after the application of a density gradient [35–38]. Although there is no direct evidence that sperm motility and sperm morphology could influence DNA density, the three characters are believed to be strongly associated[39]. Density-gradient selection of sperm can separate sperm with high motility and good morphology. Therefore, as a routine manipulation in ART, sperm separation can effectively increase the overall quality of sperm.

In addition, abnormality rates in histone to protamine transition decreased after sperm separation(Table 2). Histone to protamine transition is considered to potentially affect the sperm epigenome, and a high rate of abnormalities in protamine transition may indicate unstable sperm epigenome or immature spermatozoa[11, 40, 41]. The present study showed that protamine transition may be improved after sperm separation in semen samples. Furthermore, all subgroups of patients presented similar improvements in semen histone transition, and the rate of histone transition abnormalities decreased by 27.2% on average (Table 2). Such kind of change is likely correlated to better-quality sperm after density-gradient separation, and thus may contribute to the success of ART.

Except for changes in sperm histone transition rates, global DNA methylation decreased after sperm separation, indicating that sperm separation impacts DNA methylome stability (Fig 1). Our results demonstrated that the level of DNA methylation is lower in good-quality spermatozoa (selected sperm separated by density-gradients) than that observed in poor-quality spermatozoa (raw sperm). DNA methylation status is related to gene expression and it has been previously established that hypomethylation promotes gene expression, whereas hypermethylation inhibits gene expression. The hypomethylation status in selected sperm apparently indicates that sperm with high DNA density might have specific genes that are upregulated during spermatogenesis. Interestingly, alterations in the degree of global methylation have also been observed in elderly males [42]. Aging germ cells generally have higher rates of DNA damage and a lower capacity for reproduction. A recent studyinvolved a paired analysis of DNA from a singleindividual10–20 years apart has shown that aging sperm have a high rate of global DNA hypermethylation, and altered gene sets have been associated with specific diseases, including schizophrenia and bipolar disorder[42]. This result is consisted with our findings, as both studies have suggested that the levels of global DNA methylation are lower in good-quality spermatozoa than in poor-quality spermatozoa.

Although DNA methylation and histone retention is a critical process of spermatogenesis, we do not have direct evidence that these altered epigenetic profiles and good quality of sperm are intrinsically linked together. However, some studies have suggested that such kind of association do exist. For instance, in sperm damaged by oxidative stress, damaged DNA sequences impede the process of methylation [43]. Oxidative stress due to smoking also affects protamine protein levels and histone retention rates in mature sperm[44, 45], and aberrant methylation imprints were significantly associated with abnormal semen parameters[46].In addition, a recent study on genome-wide DNA methylation in the 90% layer (high-quality) and the 35% layer (low-quality) sperm population from single ejaculates also concluded that the low-quality sperm population displayed significantly higher variability in DNA methylation than did the high-quality sperm population[47]. The present study observed that separated, dense sperm with improved sperm quality had decreased rates of DNA methylation and histone retention (Table 2). Therefore, epigenetic alterations might not just be coincidental findings in spermatozoa selected by DNA density, but an intrinsic character of good spermatozoa itself. A spermatozoon with good morphology and motility is likely to be more genetically and epigenetically stable, thus providing adequate epigenetic information for embryonic development and offspring growth.

To investigate whether alterations in methylation after sperm selection follow a specific pattern, genome-wide analysis using a methylation array and high-throughput sequencing were conducted to detect DNA methylated and histone retention loci. All selected sperm samples showed slightly decreased rates of global methylation and 106peaks had altered methylation patterns (Table 3, Fig 2). However, we didn’t find DNA methlyation alteration in any imprinting genes region after density selection. Several studies also demonstrated that methylation of repetitive sequences such as LINE-1, Alu Yb8, NBL2 and D4Z4 altered in infertile men[46, 48]. Due to the limitation of MeDIP array, we could not confirm those changes in this study. With GO analysis, we found that the largest cluster comprised genes with different methylation status involved in gene expression and regulation.Genes such as *RCOR1*, *ZNF232*, *POU3F1*, *TCF25*, *NKX1-2*, *FOXO1*, *CITED1*, *NRIP1*, *SOX7*, *IRAK1*, *APBA1*, *KIAA0427*, *SKI*, *SQSTM1*, and *PTPRU* were hypomethylated, whereas *GATA4*, *LHX4*, *TFB2M*, and *HSF2* were hypermethylated (Table 3). These genes are known to be extensively involved in gene expression regulation and undergo significant alterations in methylation patterns during embryonic development [49, 50]. Therefore, genes for transcriptional regulation and embryonic development may have altered the methylation pattern of selected sperm.

High-throughput sequencing analysis has also detected loci with altered histone retention rates in sperm. Most genes with increased histone retention rates that were upregulated during early embryonic development were involved in neural development such as *OR4C15* and *NRXN3* (Fig 4). Further GO analysis demonstrated that after sperm separation, histones were observed to bind to a specific gene cluster that belongs to the sensory perception of smell, including *OR1C1*, *OR4C15*, *OR1K1*, *OR1J2*, *OR5L1*, *OR11H13P*, *OR5D18*, *OR11H1*, and *OR5H6* genes (Fig 3). These genes belong to the human olfactory receptor gene family, and are the large gene superfamily that encodes ORs to detect odorants[51]. The increase in the rate of histone retention of these loci might promote their expression during early embryonic development, which in turn would affect the development of the embryonic neuronal system. In addition, two genes, *ROCR1* and *APBA1*, have both been identified in sites of increased histone retention and DNA hypomethylation in selected sperm (Fig 5). *RCOR1*encodes a protein that binds to the C-terminal domain of REST and plays a specific role in neural cell differentiation[52]. APBA1is a neuronal adapter protein that interacts with amyloid precursor proteins (APPs), which have been implicated in the etiology of Alzheimer's disease [53]. Therefore, these results indicate that specific alterations on the location of histone retention might provide important information to the developing embryo and offspring, which is consisted with a recent report that the relationship between chromatin context and aberrant DNA methylation of sperm in infertile men could be locus-dependent [48].

Recent studies have suggested that specific alterations in sperm DNA can be transmitted onto the next generation, partially due to DNA methylation reprogramming and histone transition. An investigation using a mouse model showed that environmental stimuli could change the epigenetic pattern of olfactory receptor genes, which involved with an enhanced neuroanatomical representation of the Olfr151 pathway and CpG hypomethylation in some olfactory receptor genes such as Olfr151 [22]. In the present study, the genes with altered methylation and histone retention patterns were significantly clustered in the OR family, which includes 9 olfactory receptor genes that are located in different chromosomes. KEGG pathway clustering analysis indicated that the gene clusters with the highest enrichment scores were those of Alzhemier’s disease, Huntington’s disease, and Parkinson’s disease(Table G in S1 File). Although we do not know how these alterations affect embryonic development, these results indicate that genes associated with neural development are largely involved in epigenetic modification, which is in agreement with the findings of previous reports that aged sperm possess altered methylation patterns of gene associated with schizophrenia and bipolar disorder[42].

In conclusion, the present study demonstrated that sperm selection by density-gradients in ART impacts DNA epigenetic profiles. The rates of global DNA methylation and histone retention decreased in selected spermand these alterations might be locus-specific. DNA methylation changes were largely involved in transcriptional factor gene families, and histone retention decreased in numerous genes, but significantly increased in several neural development genes such as OR family. Due to the limited subjects examined by array and sequencing, our approach to identify alterations only obtain preliminary results and a causative relationship cannot be established. However, our data do raise the possibility that density selected sperm also possess different epigenetic profiles, especially for genes involved in gene transcription and neural development. These epigenetic differences in good sperm might be involved in embryonic development and offspring traits in ART.

## Supporting Information

S1 File Gene ontology (GO) and KEGG pathway cluster analysis in histone retention-associated genes. GO enrichment with positive peaks (Table A). Clustered GO enrichment with positive peaks (Table B). GO enrichment with negative peaks (Table C). Clustered GO enrichment with negative peaks (Table D). KEGG pathway enrichment with positive peaks(Table E). KEGG pathway enrichment with negative peaks(Table F). Clustered KEGG pathway enrichment with negative peaks(Table G).
(RAR)
